# Sequencing and Analysis of the Genome of *Propionibacterium freudenreichii* T82 Strain: Importance for Industry

**DOI:** 10.3390/biom10020348

**Published:** 2020-02-24

**Authors:** Kamil Piwowarek, Edyta Lipińska, Elżbieta Hać-Szymańczuk, Marek Kieliszek, Anna Maria Kot

**Affiliations:** Department of Food Biotechnology and Microbiology, Institute of Food Sciences, Warsaw University of Life Sciences - SGGW, Nowoursynowska 159 c, 02-776 Warsaw, Poland; edyta_lipinska@sggw.pl (E.L.); elzbieta_hac_szymanczuk@sggw.pl (E.H.-S.); marek_kieliszek@sggw.pl (M.K.); anna_kot@sggw.pl (A.M.K.)

**Keywords:** genome, *Propionibacterium*, metabolism, resistance, stress response

## Abstract

The genome of *Propionibacterium freudenreichii* ssp. *freudenreichii* T82, which has a chromosome containing 2,585,340 nucleotides with 67.3% GC content (guanine-cytosine content), is described in this paper. The total number of genes is 2308, of which 2260 are protein-coding genes and 48 are RNA genes. According to the genome analysis and the obtained results, the T82 strain can produce various compounds such as propionic acid, trehalose, glycogen, and B group vitamins (e.g., B6, B9, and B12). From protein-coding sequences (CDSs), genes related to stress adaptation, biosynthesis, metabolism, transport, secretion, and defense machinery were detected. In the genome of the T82 strain, sequences corresponding to the CRISPR loci (Clustered Regularly Interspaced Short Palindromic Repeats), antibiotic resistance, and restriction–modification system were found.

## 1. Introduction

Bacteria of the *Propionibacterium* genus belong to the class *Actinobacteria*, the order *Actinomycetales,* and the family *Propionibacteriaceae.* Propionic acid bacteria are divided into two groups based on the environment which they inhabit: skin (acnes) and classic (dairy). The former consists of species occurring on the skin and in the mucous membranes of the oral cavity and digestive tract, these species include *P. acnes, P. avidum, P. propionicum, P. granulosum,* and *P. lymphophilum*. Classic strains include microorganisms belonging to two phylogenetic groups. The first one includes bacteria from the species *P. acidipropionici, P. jensenii,* and *P. thoenii*. The second one includes subspecies of *P. freudenreichii* (ssp. *shermanii*, ssp. *freudenreichii*), which differ in two features: nitrate reduction and lactose fermentation abilities [1]. The strains of *P. freudenreichii* ssp. *freudenreichii* can reduce nitrates but do not have the ability to ferment lactose. In contrast, *P. freudenreichii* ssp. *shermanii* strains metabolize lactose (they have genes encoding the enzyme ß-D-galactosidase [EC 3.2.1.23]) but do not reduce nitrates. All the classic *Propionibacterium* species exhibit fermentation activity and are therefore a source of useful metabolites such as propionic acid, acetic acid, trehalose, and vitamins (B12, for instance) [2,3,4,5,6,7].

*Propionibacterium* bacteria are applied in the cheese industry, where they are used as components of inoculants (together with lactic acid fermentation bacteria that prepare the environment for the action of *Propionibacterium* strains) for the production of rennet (hard) cheeses (Swiss-Emmental, Dutch-Leerdammer, and French-Comté) and Polish semi-hard cheeses (tylzyck and krolewski). Starter cultures consisting of propionic acid bacteria (PAB) and lactic acid bacteria (*Lactobacillus plantarum, L. acidophilus, P. jensenii, and P. acidipropionici*) are also used in the production of vegetable silage. Appropriate PAB strains are also used in feed production. They are a source of vitamin B12, increase the assimilability of iron and calcium by animals, and naturally protect the product against fungal infections (propionic acid). Some of the PAB serve as probiotics in animal nutrition.

The species *P. freudenreichii* regulates the intestinal microflora by stimulating the development of *Bifidobacterium* bacteria and, through the production of bacteriocins, protects the animal organism from potential pathogens. Moreover, PAB can neutralize mycotoxins in the digestive tract, stimulate the immune system, and are a source of trehalose and vitamins: B12, B9, and K. It has been proven that the addition of PAB to the feed increases its use and the growth of young animals [8,9,10].

Some species of PAB (including *P. freudenreichii* ssp. *freudenreichii*) have the status of GRAS (Generally Recognised As Safe) and QPS (Qualified Presumption of Safety); thus, the living cells of these microorganisms and their metabolites can be used in the production of food and feed. Additionally, because PAB can produce very important compounds such as B12 vitamin, they can be used to produce vitamin B12-enriched food for vegan diets. The biomass of PAB is rich in trehalose and other vitamins from B group. This implies a huge potential for industry. As the species *P. freudenreichii* can use industrial waste for fermentation, its use in everyday life can also have a beneficial effect on the environment. To date, the complete genome sequences of *P. freudenreichii* ssp. *shermanii* CIRM-BIA1 [11] and *P. freudenreichii* ssp. *freudenreichii* DSM 20271 [12] strains have been described in the literature. To fully exploit the biotechnological potential of bacteria of the genus *Propionibacterium*, it is necessary to know their specific molecular properties, which is greatly facilitated by the knowledge of the genome. Furthermore, screening and subsequent research of another strains are also needed. Therefore, the summary classification and set of characteristics for *P. freudenreichii* T82 together with the description of the genome sequence annotation are presented below.

## 2. Materials and Methods 

### 2.1. Culture Conditions

The T82 strain was grown in VL medium consisting of 3.0 g meat extract, 10.0 g peptone, 5 g NaCl, 5 g yeast extract, 0.4 g L-cysteine hydrochloride, and 10 g glucose per liter and pH adjusted to 7.0. The cells were separated by centrifugation for 10 min at 10,000 rpm at 4 °C and washed once with sterile distilled water.

### 2.2. Genome Sequencing

Genomic DNA was isolated by CTAB/lysozyme method [13]. The quality and quantity of DNA obtained were verified by electrophoretic separation in 0.7% agarose gel and by fluorometer Qubit 2.0 (Thermo Fisher Scientific, Waltham, MA). It was mechanically fragmented with a nebulizer, and then the NGS genomic library was prepared with the KAPA Library Preparation Kit (KAPA/Roche, Basel, Switzerland). The bacterial genome library was sequenced in paired-end mode using MiSeq sequencer (Illumina, San Diego, CA) and reagents version 3 (v.3) (600 cycles). A total of 2.166.962 paired reads were obtained. Illumina sequence reads were filtered by removing poor-quality data using FastX software v.0.0.14 (http://hannonlab.cshl.edu/fastx_toolkit/). The remaining adaptor sequences were removed using Cutadapt software v.1.1 (https://github.com/marcelm/cutadapt) using default settings. The filtered data were assembled into contigs using default parameters by Newbler software v.3.0 (Roche, USA), which allowed to obtain a draft sequence of bacterial genome. Assembly metrics were generated using Quast v.5.0.2 (quast.sourceforge.net). Genome assembly resulted in generation of 58 large contigs (min. 500 bp) with a total length of 2,585,340 bp. N50 of the contigs was of 88,601 bp, the genome coverage was 211×.

### 2.3. Genome Annotation

Genes were identified using the RAST v.2.0 and KAAS v.2.2 (parameters for bacterial genome) tools—Predicted genes were translated and functionally described [14,15,16]. Metabolic pathway prediction (KEGG pathway mapping - Kyoto Encyclopedia of Genes and Genomes) was performed with RAST v.2.0 tool [14,15,16] and KAAS v.2.2 (BlastKoala) to assign KEGG Orthology (KO) numbers to each predicted CDS. Clusters of Orthologous Groups of Proteins (COGs) were determined using eggNOG v.4.5.1 [17]. Ribosomal RNA genes were detected using RNAmer v.1.2 [18] and tRNA genes were identified using tRNAscan-SE v.2.0 [19]. Genome mapping (visualization of the genome properties) was performed using CGView software v.1.0 [20]. Transmembrane helices and signal peptides were found with TMHMM v.2.0 [21] and SignalP v.5.0 [22], respectively. CRISPR loci (Clustered Regularly Interspaced Short Palindromic Repeats) were searched for using the CRISPRFinder server. Antibiotic resistance genes were predicted using Comprehensive Antibiotic Resistance Database (CARD). Furthermore, a comparison of nucleotide sequences was performed using the BLAST tool. Unless otherwise mentioned, default parameters were used.

### 2.4. Comparative Genome Analysis 

The genome sequences of *P. freudenreichii* ssp. *freudenreichii* DSM 20271 (accession no. NZ_CP010341.1) and *P. freudenreichii* ssp. *shermanii* CIRM-BIA1 (accession no. NC_014215.1) were obtained from GenBank. Sequences concerning *P. freudenreichii* ssp. *shermanii* NIZO B135 were also obtained from GenBank (accession no. AY787171.1, AY787170.1, AY787169.1). Genes were identified using the RAST v.2.0 [14,15,16]. KAAS v.2.2 (BlastKoala) was performed to assign KEGG Orthology (KO) numbers to each predicted CDS. Clusters of Orthologous Groups of Proteins (COGs) were determined using eggNOG v.4.5.1 [17].

### 2.5. Sugars Fermentation, Trehalose, and Glycogen Concentration

The ability to ferment different carbon sources by the tested strain was detected by API 50CH test, the ability to the production of trehalose (Table 1) and glycogen (Table 1) was measured by Trehalose Megazme Assay Kit and BioVision Glycogen Assay Kit, respectively. To check the ability to produce trehalose and glycogen the T82 strain was grown in medium containing apple pomace and potato wastewater (approx. 4% of sugars) in flasks in stationary conditions (30 °C) without neutralization of pH (acid stress) through the 4 days. The cells were separated by centrifugation for 10 min at 10,000 rpm at 4 °C and washed once with sterile distilled water. Then the concentration of trehalose and glycogen was measured according to the instructions attached to the kits.

### 2.6. Strain Deposition and Complete Genome Sequence Data Accession Number 

The sequence data for *P. freudenreichii* T82 genome has been deposited at GenBank under the accession number NZ_SDDY00000000.1 (Table 1).

## 3. Results and Discussion

### 3.1. General Genome Features

**Figure 1:**
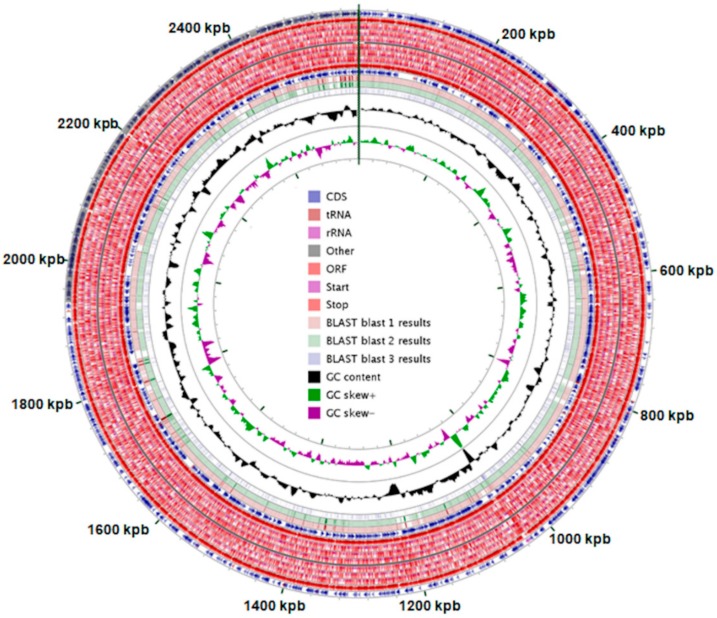
Genome properties of Propionibacterium freudenreichii ssp. freudenreichii T82 (in original size—Figure S11). Blast 1—Propionibacterium freudenreichii subsp. shermanii CIRM-BIA1, Blast 2—Propionibacterium freudenreichii subsp. freudenreichii DSM 20271, Blast 3—Tessaracoccus flavus. *genome mapping was performed using CGView software.

*Propionibacterium freudenreichii* T82 strain is a gram-positive, nonmotile, nonsporulating, mesophilic, and anaerobic to facultative anaerobic rod-shaped bacteria (Table 1). The genome of *P. freudenreichii* ssp. *freudenreichii* T82 contains 2,585,340 nucleotides with 67.3% GC content (Figure 1). The total number of genes is 2308, of which 2260 are protein-coding genes (97.9%) and 48 are RNA genes (2.08%) (Table 2). Bacteria with a small number of ribosomal operons are slow-growing organisms that can use resources efficiently and can grow under low nutrient conditions [23]. A total of 43 tRNA-encoding sequences were identified corresponding to all 20 standard amino acids (tRNAs with mismatch isotypes: (2): Ala, Gly, Pro, Thr, and Val (three sequences); Ser and Arg (4); Leu (5); Phe, Asn, Asp, His, Tyr, Trp, and Cys (1); Lys, Met, Gln, Ile, and Glu (2). The tested strain can utilize glycerol, erythritol, L-arabinose, galactose, glucose, fructose, mannose, inositol, xylitol, D-xylose, L-arabitol, potassium gluconate, esculin, and ferric citrate. The ability to ferment the last six carbon sources distinguishes the T82 strain from the DSM 20271 and CIRM-BIA1 strains (Table 3). Table 4 shows the distribution of *P. freudenreichii* T82 strain genes into functional categories according to COGs (Clusters of Orthologous Groups of Proteins) protein database [24], which groups proteins encoded by genomes of sequenced microorganisms into conservative families. These families are additionally divided into several superior functional groups.

To date, the complete sequences of *P. freudenreichii* ssp. *shermanii* CIRM-BIA1 and *P. freudenreichii* ssp. *freudenreichii* DSM 20271 strains have been described in the literature. *P. freudenreichii* T82 16S rRNA sequence shows 98% similarity with that of DSM 20271 strain. Comparison of genomes of the T82 and DSM 20271 strains revealed some other similarities. First, guanine and cytosine constitute 67.3% of all bases in the genomes of both microorganisms, whereas RNA constitutes only 2.08% of the genome. Moreover, the T82 strain can reduce nitrate to nitrite (respiratory nitrate reductase alpha–gamma chains [EC 1.7.99.4], nitrate and nitrite transporter) but cannot ferment lactose. Degradation of lactose is strain-dependent. In *P. freudenreichii* ssp. *shermanii* CIRM-BIA1 genome, the lactose locus contains three genes, namely PFREUD_02370, PFREUD_02360, and PFREUD_02350, which are responsible for encoding, respectively: β-galactosidase, LacZ; a galactosidase transporter, GalP; and an UDP-glucose isomerase, GalE1. In the *P. freudenreichii* T82 strain, sequences for only β-galactosidase were detected. All these findings indicate that the T82 strain may represent a subspecies *P. freudenreichii* ssp. *freudenreichii.*

**Figure 2:**
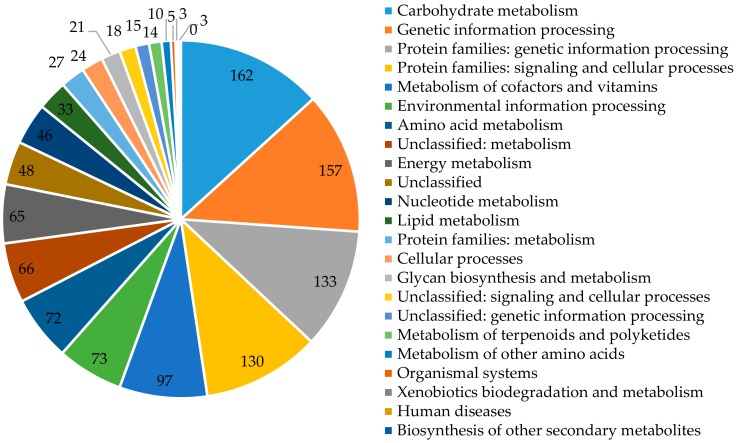
The number of genes assigned in KEGG categories.

Each group is assigned a function and contains orthological proteins from at least three phylogenetic lines, which most likely evolved from a single ancestor. Functional assignment of the examined protein is based on its classification to one or more (if it is a multidomain protein) orthologous groups on the basis of sequence similarity. For *P. freudenreichii* T82 strain, out of 2260 identified encoding sequences, 1936 sequences were grouped in 20 COGs classes (the sum of all sequences in 20 COGs classes is 2113, because some sequences are assigned to more than one class) and 1228 in KEGG categories (Figure 2).

For COGs, coding sequences were identified, inter alia, as involved in amino acid transport and metabolism (10.85%); carbohydrate transport and metabolism (8.94%); replication, recombination and repair of nucleic acids (8.41%); translation, ribosome structure, and biogenesis (7.79%); or transcription (8.21%) (Table 4). A large number of unclassified genes (KEGG-150), with unknown function (COGs-331) and hypothetical proteins (653) show that among them may be unique genes. According to COGs, the pool of genes involved in metabolism of the T82, DSM 20271 and CIRM-BIA1 strains is 952 (49.17%), 984 (48.26%), and 951 (46.94%), respectively. Regarding KEGG-(in sequence) 50.40%, 50.00%, and 50.08% of classified genes are related to categories involving metabolism (Table S1).

### 3.2. Metabolism

*Propionibacterium* bacteria need nicotinate (B3), riboflavin (B4), pantothenate (B5), biotin (B7), cobalamin (B12), and coenzyme A (CoA) for enzymatic activity and some strains additionally require thiamine (B1) [11]. Genome analysis showed that there are complete synthesis pathways of almost all these cofactors in the T82 strain (Figures S1–S3, S5, and S7), except for pantothenate (Figure S7) and biotin (Figure S9). Results described by Koskinen et al. [12] and Falentin et al. [11] also show that PAB have to be supplemented by the addition to the medium of B5 and B7 vitamins. Moreover, according to the obtained results, it is possible that the *P. freudenreichii* T82 strain can produce folate (B9 from chorismite, which is also produced by the T82 strain; Figure S10) (Figure S5), thiamine (Figure S1), and pyridoxal 5′-phosphate (active form of B6) (Figure S6). According to Deptula et al. [25], the production of active therapeutic vitamin B12 by PAB requires the synthesis of 5.6-dimethylbenzimidazole (DMBI) as the initial ligand. Genomic data indicate that *P. freudenreichii* T82 possesses a fusion gene, *bluB/cobT2* (cobalamin biosynthesis protein BluB @5.6-dimethylbenzimidazole synthase, flavin destructase family/Nicotinate-nucleotide-dimethylbenzimidazole phosphoribosyltransferase [EC 2.4.2.21]), coding for a predicted phosphoribosyltransferase/nitroreductase, which is involved in the production of DMBI and -consequently vitamin B12.

Although *P. freudenreichii* is usually cultured under anaerobic or relatively anaerobic conditions, the species, including the *P. freudenreichii* T82 strain, possess all the genes required for aerobic respiration: NADH dehydrogenase (EC 1.6.5.3, chain A-N), cytochrome complex (bd) (cytochrome d ubiquinol oxidase subunits I and II [EC 1.10.3], succinate dehydrogenase cytochrome b subunit, cytochrome c-type biogenesis protein DsbD, protein disulfide reductase [EC 1.8.1.8], putative cytochrome P450 hydroxylase) ATPase, and heme synthesis pathway (Figure S8). In anaerobic conditions, sulfates, fumarate, nitrates, and menaquinone (vitamin K2) (Figure S4) are the acceptors of electrons in *P. freudenreichii* [26].

Propionic acid bacteria produce propionic acid through the Wood–Werkman cycle. The species *P. freudenreichii* has been widely studied at the biochemical and genetic levels [11,27]. The key reaction of the Wood–Werkman cycle is the transcarboxylation reaction without free CO_2_. The enzyme catalyzing this reaction is methylmalonyl-CoA carboxytransferase, which transfers the carboxyl group from methylmalonyl-CoA to pyruvate with the formation of oxaloacetate and propionyl-CoA. This enzyme has been fully characterized. It is a biotin-dependent carboxytransferase (EC 2.1.3.1) that consists of three subunits (1,3S, 5S, and 12S). The methylmalonyl-CoA carboxytransferase is encoded by a polycistronic gene containing four coding sequences. Three of them encode the individual subunits of the enzyme, and one encodes the carrier protein transporting the carboxylic biotin. All of them are present in the *P. freudenreichii* T82 strain.

*Propionibacterium freudenreichii* shows many characteristics that allow them to survive in unfavorable environmental growth conditions. For example, they can store inorganic polyphosphate (polyP) as an energy reserve, while most bacteria store only ATP. The T82 strain also has this ability. Importantly, only bacteria that are particularly adapted to extreme conditions can use polyP as an energy source [28]. A key enzyme involved in the synthesis of this energy carrier is polyphosphate kinase (EC 2.7.4.1). The *P. freudenreichii* T82 strain also shows the presence of exopolyphosphatase (EC 3.6.1.11) and polyphosphate glucokinase (EC 2.7.1.63).

Genes potentially involved in glycogen metabolism were also identified in the genome of the T82 strain. This feature was also reported in *P. freudenreichii* ssp. *shermanii* CIRM-BIA1T strain [11]. The ability to synthesize this compound by the T82 strain depend on the sequences encoding glycogen synthase (EC 2.4.1.21), glycogen phosphorylase (EC 2.4.1.1), and enzymes branching glycogen (EC 2.4.1.18). Some of these genes were also found in *P. acnes*. Because *P. freudenreichii* and *P. acnes* cannot ferment extracellular glycogen, these enzymes are expected to be involved in intracellular glycogen accumulation and/or hydrolysis.

Strains of *P. freudenreichii* [29], including T82, can also synthesize and accumulate trehalose from glucose and pyruvate. The synthesis of trehalose by *Propionibacterium* occurs at the beginning of the stationary phase under oxidative, osmotic, and acidic stress conditions. Trehalose is synthesized by PAB through the synthase pathway of trehalose-6-phosphate (EC 2.4.1.15)/phosphatase (EC 3.1.3.12) (OtsA-OtsB) and catalyzed by trehalose synthase (TreS) (EC 5.4.99.16). The *otsA, otsB*, and *treS* genes were previously identified in NIZO B365, DSM 20271, and CIRM-BIA1 strains. In the *P. freudenreichii* T82 strain, nucleotide sequences encoding these three genes are similar to those reported by Cardoso et al. [29], Koskinen et al. [12], and Falentin et al. [11], respectively: 98.45% (NIZO B365) and 100% (DSM and CIRM-BIA1); 99.07% (NIZO B365), 99.88% (DSM), and 99.65% (CIRM-BIA1); and 99.78% (NIZO B365), 99.78% (DSM 20271), and 99.89% (CIRM-BIA1). 

### 3.3. Resistance and Stress Response

The biosynthesis of propionic acid by *Propionibacterium* is inhibited mainly by a negative feedback mechanism and stress conditions. According to the RAST analysis, in theT82 strain, 57 genes are responsible for stress response and are divided into six groups: osmotic (7), oxidative (26), cold shock (2), heat shock (15), detoxification (10), and no subcategory (4) (Table S2). Therefore, as suggested by researchers, the most effective strategy to increase PAB biomass and propionic acid synthesis is improving the resistance of these bacteria to low pH and stress conditions in general. For this purpose, adaptive evolution and genome shuffling have been used [30]. It was also found that arginine deaminase (EC 3.5.3.6) and glutamate decarboxylase (EC 4.1.1.15) (the sequence encoding this gene is present in the genome of the T82 strain) play an important role in acid tolerance of *P. acidipropionici* [31,32]. Guan et al. [33] attempted to improve the resistance of *P. jensenii* ATCC 4868 strain to low pH by inducing overexpression of five genes: *Arca, ARCC, gadB, GDH,* and *ybaS*. Suwannakham et al. [34] removed the *ack* gene encoding acetic kinase from the genome of *P. acidipropionici* strain, thus increasing propionic acid production. Immobilization was also used to increase PAB resistance to stress conditions [35]. However, the current knowledge on the functioning of acid resistance in the cells of *Propionibacterium* remains at the microenvironmental level. Therefore, further research is needed to understand these mechanisms. System biology methods and genome analysis may be useful in this context. Technologies comparing genomics and transcriptomics can be used to induce resistance of strains to acids at the DNA and RNA level, while proteomics and metabolomics can be used to identify key proteins and metabolites as well as the pathways responsible for a particular trait. For example, Lu et al. [36] identified a previously unknown system affecting the acid resistance of *Escherichia coli*, namely the transformation of L-glutamine into L-glutamic acid with the simultaneous production of ammonia, alkalizing the culturing environment. 

As the examples show, although it is possible to increase the yield of propionic acid by bacteria of the *Propionibacterium* genus, this task is quite difficult. The inhibitors of metabolic and genetic engineering of *Propionibacterium* are their restriction–modification (RM) systems [37,38]. These systems provide protective mechanisms against foreign DNA and bacteriophages, which, at the same time, are an obstacle to genetic manipulation. To distinguish foreign DNA from the host DNA, these systems coordinate the action of restrictive and modifying enzymes, thus protecting *Propionibacterium* cells from the penetration of foreign genetic material. In classical RM systems, foreign DNA is cleaved or restricted by endonucleases. Host cell DNA avoids restriction through the methylation, or modification, of certain adenine or cytosine residues in the target sequence. On the basis of subunit composition, cofactor requirements, and position of the DNA cleavage site, RM systems have been classified into four distinct groups, namely, type I, type II, type III, and type IV. In the *P. freudenreichii* T82 strain, the type I RM system was detected (type I restriction-modification system, DNA-methyltransferase subunits M, R, and S [EC 2.1.1.72], type I restriction-modification system, restriction subunit R [EC 3.1.21.3]) and two sequences involved in type III (type III restriction-modification system methylation subunit [EC 2.1.1.72] and type III restriction-modification enzyme helicase subunit). The type I RM system is a bifunctional, multisubunit complex containing products of the *hsdR*, *hsdM*, and *hsdS* genes (host specificity for DNA) [39]. HsdS interacts with the target sequence as a component of the restriction and modification complexes. HsdS consists of four domains: two variable target recognition domains (TRDs), a central-conserved domain, and a conserved C-terminus domain. The type I target sequence is asymmetric and composed of two half-sites: a 5ʹ half-site of 3–4 bp and a 3ʹ half-site of 4–5 bp, separated by a nonspecific spacer of 6–8 bp. In the HsdS subunit, each TRD recognizes one half-site, while the conserved domains are thought to interact with the HsdR and HsdM proteins in the complex. 

CRISPR represent a family of DNA repeats providing acquired immunity against foreign genetic elements, for example, protection against bacteriophages [40]. They consist of short and highly conserved repeats with variable sequences called spacers. CRISPR-associated genes (*Cas*) are found often next to these sequences. CRISPR are found only in 40% of bacterial genomes [40]. The genome of the *P. freudenreichii* T82 strain is composed of three confirmed and two questionable CRISPR loci (Table S3). The detected CRISPR/CRISPR-associated (Cas) system consists of three Cas genes: Cas1, Cas2, and Cas4. 

The CRISPR1 locus, located between 16 and 3173 bp (Scaffold 16—length 59,854 bp), harbors a 36 bp direct repeat (DR) sequence GCCTCAATGAAGGGCCCCTCCAGAAGGAGGGGCAAT and 43 spacer sequences. Blast results on the EMBL phage database showed that the 10 spacer show a strong similarity to the *Propionibacterium* phages - spacer 9 (PFR2 38/38 nt, PFR1 38/38 nt), spacer 17 (PFR2 39/39 nt, PFR1 39/39 nt), spacer 23 (PFR2 38/38 nt, PFR1 38/38 nt), 27 spacer (E6 38/38 nt, Doucette 38/38 nt), spacer 28 (E6 40/40 nt, Doucette 40/40 nt, B22 40/40 nt), spacer 29 (Doucette 35/35 nt), spacer 33 (G4 37/37 nt), spacer 38 (G4 37/37 nt, E1 37/37 nt, B22 37/37 nt, B3 37/37 nt, Anatole 37/37 nt, spacer 39 (G4 35/35 nt), spacer 43 (Doucette 37/37 nt). Two other possible CRISPR loci were identified (Table S3). The presence of CRISPR loci causes that the genome stability of a bacterial strain may be increasing, therefore, also its adaptation to the environment. What is more, it strongly suggests that *P. freudenreichii* T82 has had a contact with phages—they may contribute to its resistance to phage attacks [11].

RNA modification enzymes manifested as methyltransferases play a large role in antibiotic resistance in bacteria. The modifications of the ribosome almost exclusively involve methylation of various positions on the bases or at the 2’-O-ribose position. Extensive information on antibiotic resistance caused by methylation of rRNA is available. Modifications at eight 23S rRNA nucleotides (G748, A1067, C1920, A2058, G2470, U2479, A2503, and G2535) on the large ribosomal subunit have thus far been revealed as antibiotic resistance determinants [41]. In the *P. freudenreichii* T82 strain, the 23S rRNA with mutations at G2294A and G2295A is responsible for resistance to macrolide antibiotics. This shows that this strain had direct contact with the environment in which antibiotics were present, probably soil or cattle, which is one of the most common sources of *P. freudenreichii*.

Some strains of *Propionibacterium* are able to accumulate glycine betaine which is involved in long-term survival by acting as a chemical chaperone [11]. Genes supporting glycine betaine transport were identified in the genome of *P. freudenreichii* T82. On the other hand, genes responsible for encoding enzymes engaged in producing this compound were not detected—betaine aldehyde dehydrogenase (EC 1.2.1.8) and choline dehydrogenase (EC 1.1.99.1). It is possible that these genes are amongst uncharacterized coding sequences (Table S4).

Regulation of intracellular pH is crucial for survival. Analysis of the *P. freudenreichii* T82 strain genome shows a complete *atp*BEFHAGDC operon with gamma chain (*atp*G, PFREUD_10480) which is activated in *P. freudenreichii* by acid and bile salts. To keep intracellular pH homeostasis the genome of the T82 strain contains genes encoding: pyruvate-flavodoxin oxidoreductase, aspartate ammonia-lyase (EC 4.3.1.1), glutamate decarboxylase (EC 4.1.1.15), and succinate dehydrogenase (Table S4). 

The genome of the *P. freudenreichii* T82 strain was found to encode few proteins involved in transcriptional regulation, including three genes encoding sigma factors and 59 genes responsible for transcriptional regulators. First, a relatively large number of regulatory proteins are present in the large genomes; this probably confirm that this strain has the machinery to adapt to different environmental niches. Regulatory proteins are important for the adaptation of an organism to different environments [42]. The T82 strain carries various genes encoding heat stress proteins, namely *DnaJ, DnaK, GrpE, GroES,* and *GroEL,* and cold stress proteins from the CSP family. The genes in the T82 strain genome may also be involved in oxidative stress tolerance by coding for proteins such as thioredoxin reductase, peroxidase, catalase, and superoxide dismutase. Bacteria of the *P. freudenreichii* genus possesses a lot of genes for disulfide-reduction and to eliminate of reactive oxygen forms (cysteine synthase, glutathione S-transferase, omega) [43]. Genes involved in the redox-dependent regulation of nucleus processes are also found. The similar situation was found for the other strains of bacteria of the *Propionibacterium* genus (Table S4).

Among the genes activated in response to stressors are polybibonucleotide nucleotidyltransferase and inosine dehydrogenase—it suggests the ability of the T82 strain to synthesize alarmon ppGpp during the stress conditions (Table S4). 

## 4. Conclusions

The presence of genes involved in the metabolism of phosphates, glycogen, trehalose, and CRISPR loci and the genes responsible for stress response in the genome of *P. freudenreichii* T82 strain make this strain well adapted to the culturing environment (as shown by the results of this study) and capable of long-term survival under culturing conditions, especially in the stationary phase. These properties along with the ability of this strain to produce valuable metabolites indicate that this strain has a huge potential in the industry for the production of propionic acid, vitamins, and trehalose and for product enrichment with the biomass of PAB. For this purpose, further research is needed to increase cost-effectiveness and achieve high production efficiencies of specific metabolites. The first condition can be met by using industrial waste as microbiological media, which contain in its composition nutrient sources assimilable by a given strain. Production efficiency can be improved using metabolic and genetic engineering tools. Furthermore, knowledge of the genetic material of the strain used at the molecular level (matching the strain to a specific waste and improvement of the strain through genetic modification) will certainly help in the development of technological processes.

## Figures and Tables

**Table 1:** Classification and general features of *Propionibacterium freudenreichii* subspecies *freudenreichii* T82 according to the MIGS recommendations (Minimum Information about a Genome Sequence) [12].

MIGS ID	Property	Term
	Classification	**Domain** Bacteria **Phylum** *Actinobacteria* **Class** *Actinobacteria* **Order** *Propionbacteriales* **Family** *Propionibacteriaceae* **Genus** *Propionibacterium* **Species** *Propionibacterium freudenreichii* **subspecies** *freudenreichii*
	GenBank accession no.	NZ_SDDY00000000.1
	Bioproject (NCBI)	PRJNA224116
	Biosample (NCBI)	SAMN10768998
	Gram strain	Positive
	Cell shape	Rod
	Motility	No
	Sporulation	No
	Optimum temperature	37°C
	Optimum pH	approx. 7.0
	Trehalose	+/(50.91 mg/g d.m.) (Megazyme test)
	Glycogen	+/(166.07 µg/g d.m.) (BioVision Glycogen)
**MIGS-6**	Habitat	Unknown
**MIGS-6.3**	Salinity	Unknown
**MIGS-22**	Oxygen-requirement	Anaerobic/facultative anaerobic
**MIGHS-15**	Biotic relationship	Free-living
**MIGS-14**	Phatogenicity	Non-pathogen
**MIGS-4**	Geographic location	Unknown
**MIGS-5**	Sample collection	Unknown
**MIGS-4.1**	Latitude	Unknown
**MIGS-4.2**	Longitude	Unknown
**MIGS-4.4**	Altitude	Unknown

**Table 2:** Genome statistics.

Attribute	Value	% of Total
Genome size (bp)	2,585,340	100.00
DNA G + C (bp)	1.739.933	67.30
Total genes	2308	100.00
Protein coding genes	2260	97.90
RNA genes	48	2.08
tRNA	45	1.95
rRNA	3	0.12
Genes assigned to COGs	1936	85.66
Genes assigned to KEGG	1222	49.64
Gen with transmembrane helices	665	28.81
Genes with signal peptides	142	6.15

**Table 3:** *Propionibacterium freudenreichii* carbon substrate degradation.

Carbon Source	*P. freudenreichii* T82	*P. freudenreichii* DSM 20271 [12]	*P. freudenreichii* CIRM-BIA1 [11]
Glucose	+	+	+
Fructose	+	+	+
Mannose	+	-	+
Glycerol	+	+	+
Adonitol	-	+	+
Inositol	+	+	+
Erythritol	+	+	+
Galactose	+	+	+
Lactose	-	-	+
Lactic acid	No data	-	+
Gluconic acid	No data	-	-
Esculine hydrolisis	No data	-	+
L-arabinose	+	+	+
Ribose	-	-	-
Melbiose	-	-	-
Raffinose	-	-	-
Saccharose	-	-	-
L-arabitol	+	-	-
Xylitol	+	-	-
D-xylose	+	-	-
Esculin	+	-	-
Ferric citrate	+	-	-
Potassium gluconate	+	-	-

**Table 4:** Number of genes associated with general Clusters of Orthologous Groups of Proteins (COGs) functional categories.

Code	Description	Value ^1^	%	Value ^2^	%	Value ^3^	%
	Information Storage and Processing	T82		DSM 20271 [12]		CIRM-BIA1 [11]	
**J**	Translation, ribosomal structure and biogenesis	151	7.79	153	7.50	155	7.65
**A**	RNA processing and modification	0	0.00	0	0.00	0	0.00
**K**	Transcription	159	8.21	159	7.80	165	8.14
**L**	Replication, recombination and repair	163	8.41	227	11.13	242	11.94
**B**	Chromatin structure and dynamics	0	0.00	0	0.00	0	0.00
	**Cell processes and signaling**						
**D**	Cell cycle control, Cell division, chromosome partitioning	30	1.55	30	1.47	32	1.58
**Y**	Nuclear structure	0	0.00	0	0.00	0	0.00
**V**	Defense mechanisms	39	2.01	40	1.96	42	2.07
**T**	Signal transduction mechanisms	66	3.41	69	3.24	69	3.41
**M**	Cell wall/membrane biogenesis	100	5.17	99	4.86	95	4.69
**N**	Cell motility	9	0.04	9	0.44	9	0.44
**Z**	Cytoskeleton	0	0.00	0	0.00	1	0.05
**W**	Extracellular structures	0	0.00	0	0.00	0	0.00
**U**	Intracellular trafficking and secretion	39	2.01	37	1.81	35	1.73
**O**	Posttranslational modification, protein turnover, chaperones	74	3.82	78	3.82	81	4.00
	**Metabolism**						
**C**	Energy production and conversion	136	7.02	141	6.91	140	6.91
**G**	Carbohydrate transport and metabolism	173	8.94	174	8.53	174	8.59
**E**	Amino acid transport and metabolism	210	10.85	221	10.85	214	10.56
**F**	Nucleotide transport and metabolism	65	3.36	65	3.19	64	3.16
**H**	Coenzyme transport and metabolism	113	5.84	116	5.70	112	5.52
**I**	Lipid transport and metabolism	64	3.31	65	3.19	64	3.16
**P**	Inorganic ion transport and metabolism	153	7.90	154	7.55	140	6.91
**Q**	Secondary metabolites biosynthesis, transport and catabolism	38	1.96	48	2.35	43	2.12
	**Uncharacterized**						
**R**	General function prediction	0	0.00	0	0.00	0	0.00
**S**	Function unknown	331	17.09	336	16.48	331	16.34

^1^ genes assigned to COGs—1936 (the total value is 2113, some genes were assigned to more than one class); ^2^ genes assigned to COGs—2039 (the total value is 2221, some genes were assigned to more than one class); ^3^ genes assigned to COGs—2026 (the total value is 2078, some genes were assigned to more than one class); green—the same numeric values/orange—the same percentages/white—different values.

## Supplementary Materials

The following are available online at https://www.mdpi.com/2218-273X/10/2/348/s1.
