# Supplementary material for: Sequencing and Analysis of the Genome of Propionibacterium freudenreichii T82 Strain: Importance for Industry
Source: Biomolecules. 2020 Feb 24;10(2):348. doi: 10.3390/biom10020348 (PMC7072396; doi:10.3390/biom10020348)
Supplement: Supplementary file 1 [file biomolecules-10-00348-s001.zip › Supplementary materials/Supplementary material.docx]

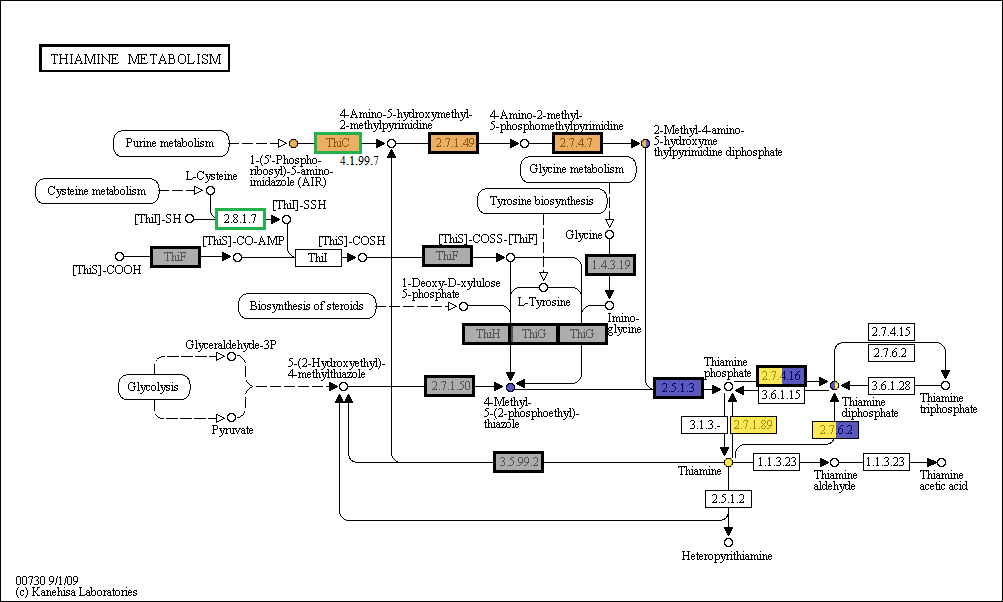


**2.7.4.3**

4.1.99.17

**Figure S1.** Thiamine metabolism in *P. freudenreichii* T82

black and green frames - genes involved in metabolism.

Generated by RAST.


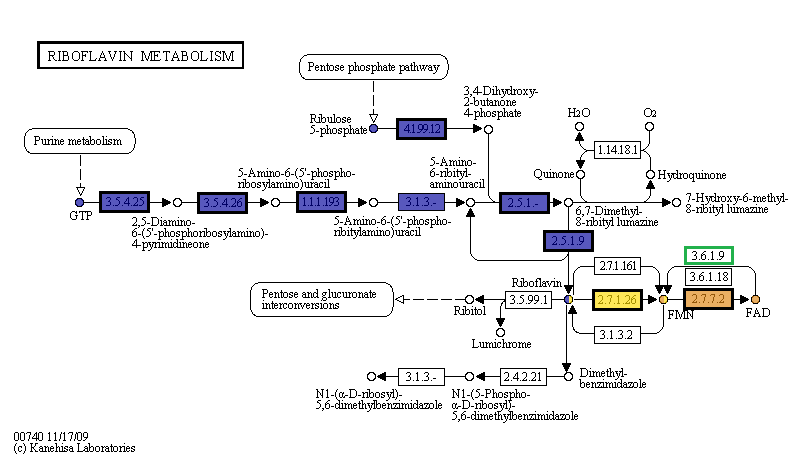


**1.5.1.38**

**1.13.11.79**

**3.1.3.104**

**Figure S2.** Riboflavin metabolism in *P. freudenreichii* T82

black and green frames - genes involved in metabolism.

Generated by RAST.


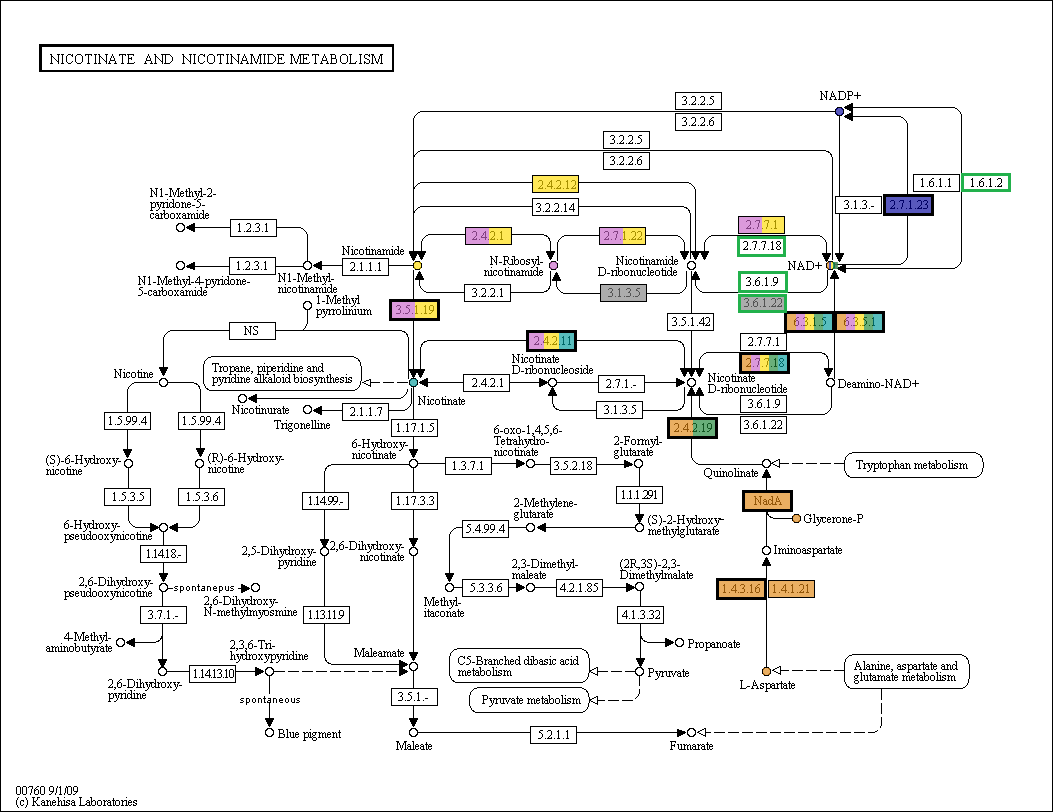


**2.3.1.286**

**Figure S3.** Nicotinate metabolism in *P. freudenreichii* T82

black and green frames - genes involved in metabolism.

Generated by RAST.


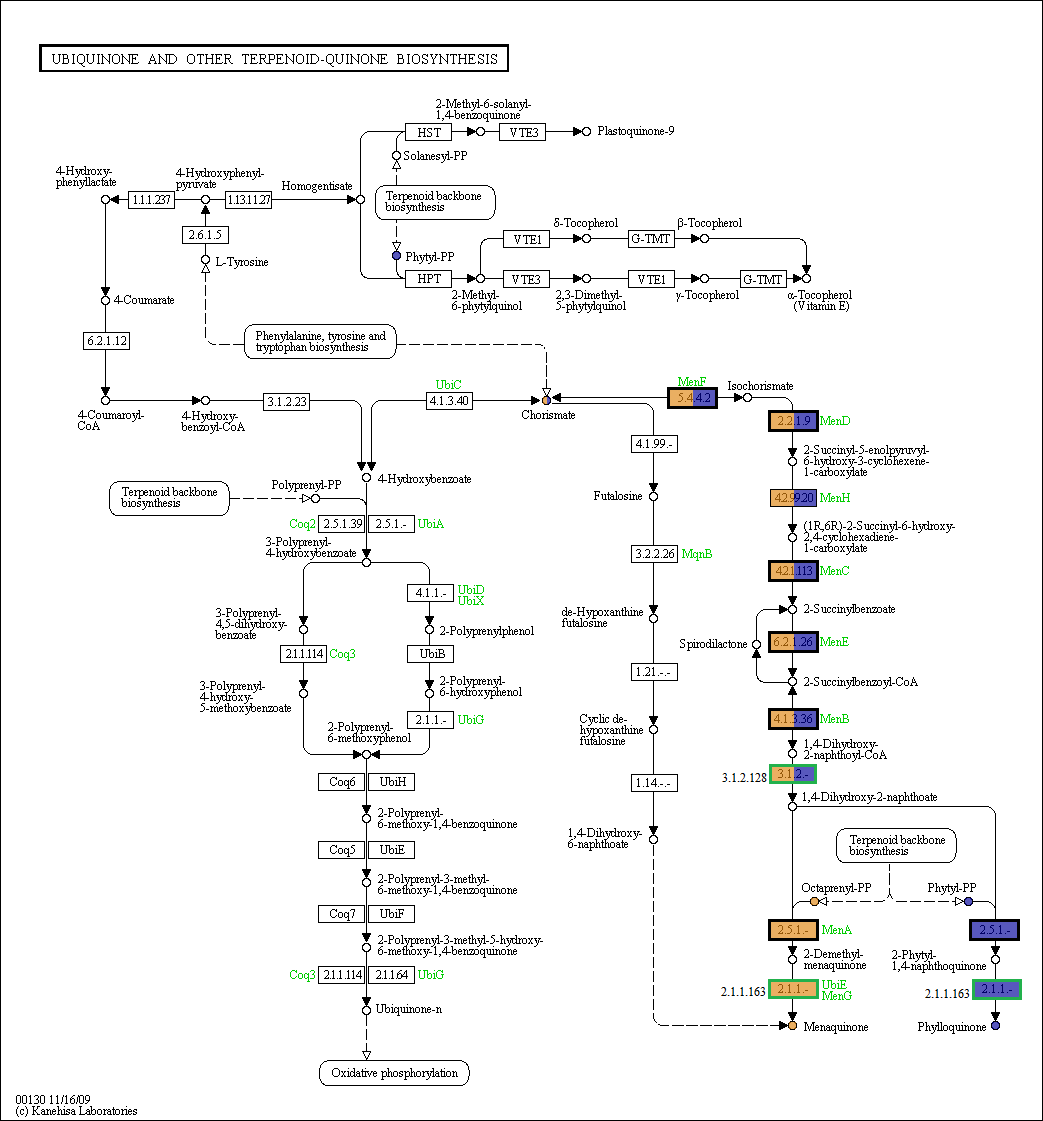


3.1.2.28

**Figure S4.** Vitamin K2 metabolism in *P. freudenreichii* T82

black and green frames - genes involved in metabolism.

Generated by RAST.


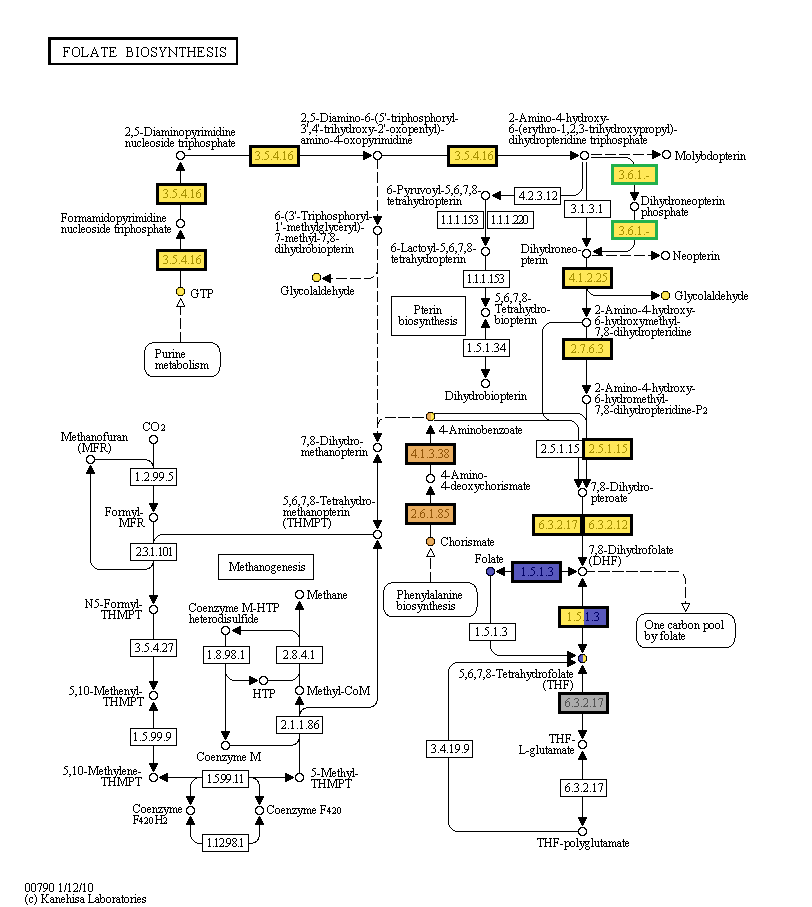


**Figure S5.** Folate metabolism in *P. freudenreichii* T82

black and green frames - genes involved in metabolism.

Generated by RAST.


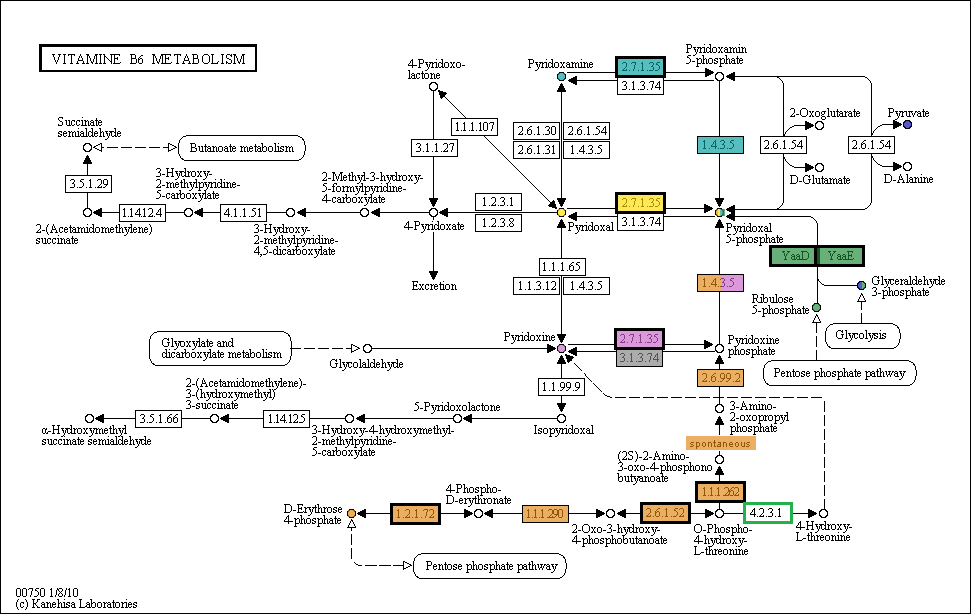


**Figure S6.** Vitamin B6 metabolism in *P. freudenreichii* T82

black and green frames - genes involved in metabolism.

Generated by RAST.


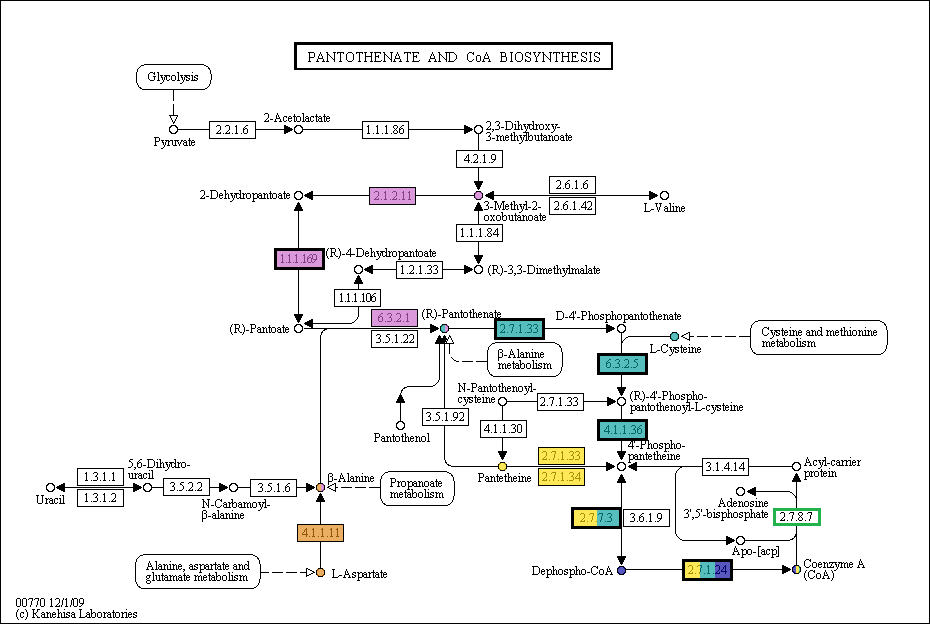


**Figure S7.** Pantothenate and CoA metabolism in *P. freudenreichii* T82

black and green frames - genes involved in metabolism.

Generated by RAST.


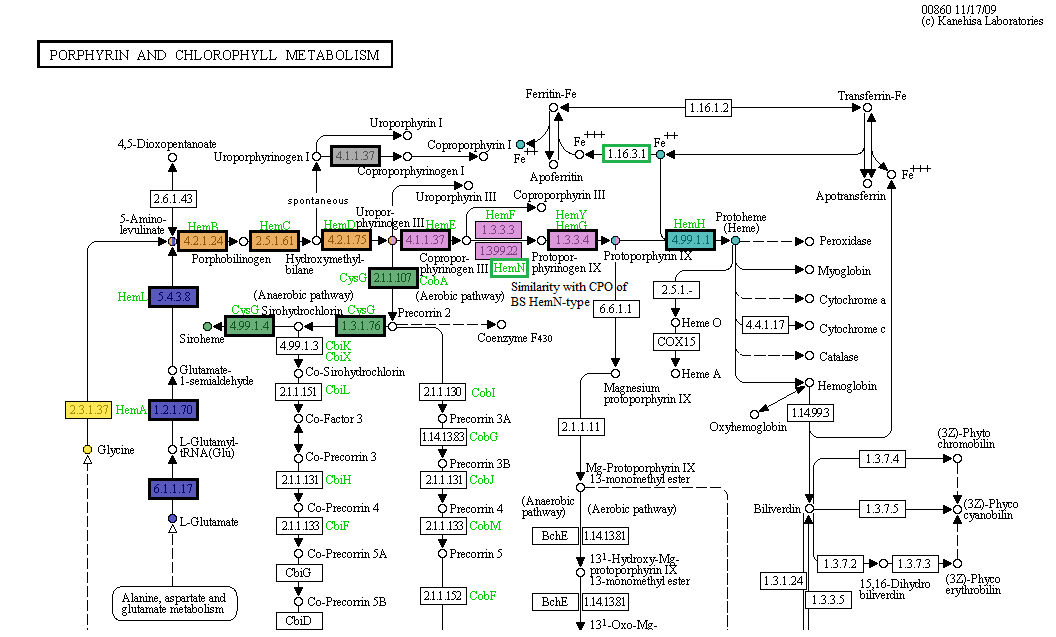


**Figure S8.** Heme synthesis pathway in *P. freudenreichii* T82

black and green frames - genes involved in metabolism.

Generated by RAST.


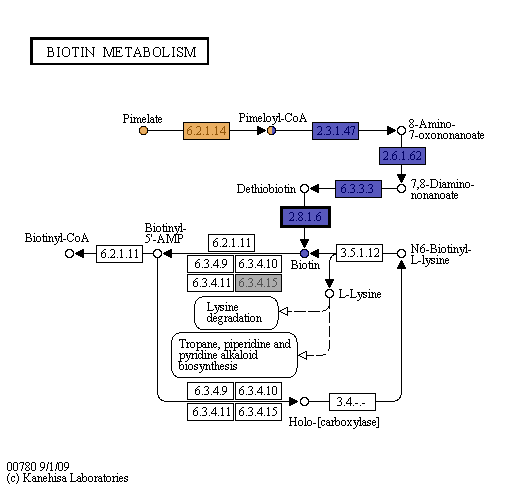


**Figure S9.** Biotin metabolism in *P. freudenreichii* T82

black and green frames - genes involved in metabolism.

Generated by RAST.


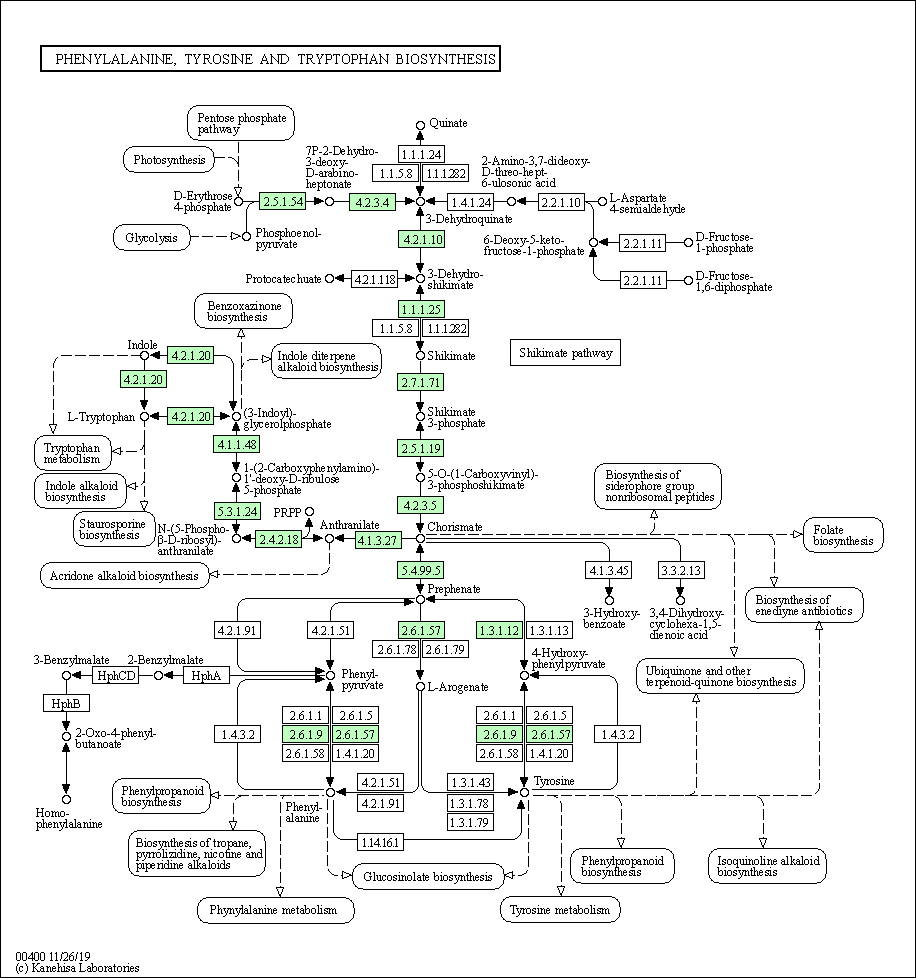


**Figure S10.** Chorismate metabolism in *P. freudenreichii* T82

green - genes involved in metabolism.

Generated by KAAS BlastKoala.

**Table S1.** The number of genes assigned in KEGG categories – comparison with the other strains.

| KEGG categories | T82 | DSM 20271 | CIRM-BIA1 |
| --- | --- | --- | --- |
| Carbohydrate metabolism | 162 | 162 | 161 |
| Genetic information processing | 157 | 161 | 163 |
| Protein families: genetic information processing | 133 | 137 | 136 |
| Protein families: signaling and cellular processes | 130 | 135 | 131 |
| Metabolism of cofactors and vitamins | 97 | 100 | 97 |
| Environmental information processing | 73 | 76 | 72 |
| Amino acid metabolism | 72 | 75 | 74 |
| Unclassified: metabolism | 66 | 73 | 72 |
| Energy metabolism | 65 | 66 | 61 |
| Unclassified | 48 | 48 | 47 |
| Nucleotide metabolism | 46 | 47 | 47 |
| Lipid metabolism | 33 | 33 | 33 |
| Protein families: metabolism | 27 | 26 | 28 |
| Cellular processes | 24 | 24 | 21 |
| Glycan biosynthesis and metabolism | 21 | 21 | 20 |
| Unclassified: signaling and cellular processes | 18 | 21 | 18 |
| Unclassified: genetic information processing | 15 | 17 | 18 |
| Metabolism of terpenoids and polyketides | 14 | 14 | 14 |
| Metabolism of other amino acids | 10 | 10 | 10 |
| Organismal systems | 5 | 6 | 6 |
| Xenobiotics biodegradation and metabolism | 3 | 1 | 1 |
| Human diseases | 3 | 2 | 4 |
| Biosynthesis of other secondary metabolites | 0 | 1 | 0 |
| Total | 1222 | 1256 | 1234 |

green - the same values selected/white – different values

**Table S2.** Categories of stress response in *P. freudenreichii* T82.

| Osmotic stress | Oxidative stress | Cold shock | Heat shock | Stress Response - no subcategory |
| --- | --- | --- | --- | --- |
| [Osmoregulation](http://rast.theseed.org/FIG/seedviewer.cgi?page=Subsystems&subsystem=Osmoregulation&organism=6666666.333287) (1) | [Protection from Reactive Oxygen Species](http://rast.theseed.org/FIG/seedviewer.cgi?page=Subsystems&subsystem=Protection_from_Reactive_Oxygen_Species&organism=6666666.333287) (1) | [Cold shock, CspA family of proteins](http://rast.theseed.org/FIG/seedviewer.cgi?page=Subsystems&subsystem=Cold_shock,_CspA_family_of_proteins&organism=6666666.333287) (2) | [Heat shock](http://rast.theseed.org/FIG/seedviewer.cgi?page=Subsystems&subsystem=Heat_shock_dnaK_gene_cluster_extended&organism=6666666.333287)  (11) | [SigmaB stress responce regulation](http://rast.theseed.org/FIG/seedviewer.cgi?page=Subsystems&subsystem=SigmaB_stress_responce_regulation&organism=6666666.333287) (3) |
| [Choline and Betaine Uptake and Betaine Biosynthesis](http://rast.theseed.org/FIG/seedviewer.cgi?page=Subsystems&subsystem=Choline_and_Betaine_Uptake_and_Betaine_Biosynthesis&organism=6666666.333287) (6) | [Oxidative stress](http://rast.theseed.org/FIG/seedviewer.cgi?page=Subsystems&subsystem=Oxidative_stress&organism=6666666.333287) (10) |  |  | [Hfl operon](http://rast.theseed.org/FIG/seedviewer.cgi?page=Subsystems&subsystem=Hfl_operon&organism=6666666.333287) (1) |
|  | [Glutathione analogs: mycothiol](http://rast.theseed.org/FIG/seedviewer.cgi?page=Subsystems&subsystem=Glutathione_analogs:_mycothiol&organism=6666666.333287) (7) |  |  |  |
|  | [Glutathione: Non-redox reactions](http://rast.theseed.org/FIG/seedviewer.cgi?page=Subsystems&subsystem=Glutathione:_Non-redox_reactions&organism=6666666.333287) (3) |  |  | [Carbon Starvation](http://rast.theseed.org/FIG/seedviewer.cgi?page=Subsystems&subsystem=Carbon_Starvation&organism=6666666.333287) (1) |
|  | [Redox-dependent regulation](http://rast.theseed.org/FIG/seedviewer.cgi?page=Subsystems&subsystem=Redox-dependent_regulation_of_nucleus_processes&organism=6666666.333287) (5) |  |  |  |

based on RAST

**Table S3.** CRISPR loci detected in *P. freudenreichii* T82 strain.

| CRISPR | Start | End | Length | DR length | Number of spacer | DR consensus |
| --- | --- | --- | --- | --- | --- | --- |
| Confirmed | | | | | | |
| CRIPSR 1 Scaffold 16  length 59854 bp | 16 | 3173 | 3157 | 36 | 43 | GCCTCAATGAAGGGCCCCTCCAGAAGGAGGGGCAAT |
| CRIPSR 2 Scaffold 44  length 9600 bp | 1 | 322 | 321 | 32 | 4 | CCCCTCCTTCTGGAGGGGCCCTTCATTGAGGC |
| CRIPSR 3 Scaffold 52  length 3076 bp | 2510 | 3045 | 535 | 36 | 7 | GCCTCAATGAAGGGCCCCTCCAGAAGGAGGGGCAAT |
| Questionable | | | | | | |
| CRIPSR Scaffold 16  length 59854 bp | 37198 | 37333 | 135 | 25 | 2 | GGGACAAGGCCGTGGGGGACAAGGC |
| CRISPR Scaffold 24  length 40951 bp | 6252 | 6346 | 94 | 21 | 1 | AACGGACGCGTCGGCTCATCCGA |

**Table S4.** Selected genes involved in resistance and stress response – comparison with the other strains.

|  | T82 | | | | DSM 20271 | | | | CIRM-BIA1 | | | |
| --- | --- | --- | --- | --- | --- | --- | --- | --- | --- | --- | --- | --- |
| CRISPR loci  (confirmed) | Nr | DR length | Number of spacer | DR consensus | Nr | DR length | Number of spacer | DR consensus | Nr | DR length | Number of spacer | DR consensus |
|  | 1 | 36 | 43 | GCCTCAATG  AAGGGCCC  CTCCAGAAGG  AGGGGCAAT | 1 | 36 | 41 | GCCTCAATGA  AGGGCCCCTC  CAGAAGGAGG  GGCAAT | 1 | 36 | 33 | GCCTCAATGA  AGGGCCCCT  CCAGAAGGAGGG  GCAAT |
|  | 2 | 32 | 4 | CCCCTCCTTCT  GGAGGGGCCC  TTCATTGAGGC | 2 | 36 | 23 | GCCTCAATG  AAGGGCCCC  TCCAGAAGGA  GGGGCAAT |  | | | |
|  | 3 | 36 | 7 | GCCTCAATGA  AGGGCCCCTCC  AGAAGGAGGG  GCAAT |  | | | |  | | | |
|  | 2 questionable loci | | | | 4 questionable loci | | | | 4 questionable loci | | | |
| RM system | **13 coding sequences**  Type I restriction-modification system, DNA-methyltransferase subunit M (EC 2.1.1.72)  Type III restriction-modification system methylation subunit (EC 2.1.1.72)  Type III restriction-modification enzyme helicase subunit  Type I restriction-modification system, restriction subunit R (EC 3.1.21.3)  Type I restriction-modification system, specificity subunit S  Type I restriction-modification system, specificity subunit S  Type I restriction-modification system, DNA-methyltransferase subunit M (EC 2.1.1.72)  Type I restriction-modification system, restriction subunit R (EC 3.1.21.3)  Type I restriction-modification system, DNA-methyltransferase subunit M (EC 2.1.1.72)  Type I restriction-modification system, restriction subunit R (EC 3.1.21.3)  Type I restriction-modification system, specificity subunit S  Type I restriction-modification system, DNA-methyltransferase subunit M (EC 2.1.1.72)  Type I restriction-modification system, DNA-methyltransferase subunit M (EC 2.1.1.72) | | | | **6 coding sequences**  Type I restriction-modification system, restriction subunit R (EC 3.1.21.3)  Type I restriction-modification system, specificity subunit S  Type I restriction-modification system, DNA-methyltransferase subunit M (EC 2.1.1.72)  Type III restriction-modification system restriction subunit (EC 3.1.21.5)  Type III restriction-modification system methylation subunit (EC 2.1.1.72)  Type III restriction-modification system methylation subunit (EC 2.1.1.72) | | | | **7 coding sequences**  Type I restriction-modification system, restriction subunit R (EC 3.1.21.3) Restriction modification system DNA specificity domain  Type I restriction-modification system, specificity subunit S  Type I restriction-modification system, DNA-methyltransferase subunit M (EC 2.1.1.72)  Type I restriction-modification system, DNA-methyltransferase subunit M (EC 2.1.1.72)  Type III restriction-modification system methylation subunit (EC 2.1.1.72)  Type III restriction-modification enzyme helicase subunit | | | |
| Transcriptional regulator | **62 coding sequences**  Transcriptional regulator, AsnC family Transcriptional regulator  Transcriptional regulator, GntR family Transcriptional regulator, LacI family Transcriptional regulator, MerR family Transcriptional regulator  Transcriptional regulator, PadR family Putative transcriptional regulator Periplasmic binding protein/LacI transcriptional regulator  Transcriptional regulator, MerR family Transcriptional regulator, AcrR family Transcriptional regulator, AcrR family Transcriptional regulator, MarR family Transcriptional regulator, RpiR family Transcriptional regulator, GntR family Transcriptional regulator, AcrR family Putative TetR-family transcriptional regulator  Phosphate regulon transcriptional regulatory protein PhoB (SphR) CarD-like transcriptional regulator  Respiratory nitrate reductase gamma chain (EC 1.7.99.4) / Transcriptional regulator, ArsR family / Molybdopterin synthase sulfur carrier subunit Transcriptional regulator, AcrR family Transcriptional regulator, ArsR family Transcriptional regulator, MerR family Transcriptional regulator, GntR family Mn-dependent transcriptional regulator MntR  Transcriptional regulator, LacI family Transcriptional regulator  Ribonucleotide reductase transcriptional regulator NrdR  Transcriptional regulator, Xre family Transcriptional regulator, PadR family Transcriptional regulator, AcrR family Transcriptional regulator, ArsR family Transcriptional regulator, AcrR family Transcriptional regulator, GntR family Transcriptional regulator, MarR family Two component sensory transduction transcriptional regulatory protein MtrA Transcriptional regulator, ArsR family Transcriptional regulator, AcrR family Transcriptional regulator, AcrR family Transcriptional regulator, ArsR family regulatory protein, LacI:Periplasmic binding protein/LacI transcriptional regulator  Transcriptional regulator, AcrR family Putative AsnC-family transcriptional regulatory protein  Nudix-related transcriptional regulator NrtR  FIG005453: Putative DeoR-family transcriptional regulator  Transcriptional regulator, IclR family Transcriptional regulator, MarR family Transcriptional regulator, ArsR family Transcriptional regulator, AcrR family Transcriptional regulatory protein PadR Transcriptional regulator, AcrR family Transcriptional regulator, MarR family Transcriptional regulator, DeoR family Transcriptional regulator MarR Transcriptional regulator, Crp/Fnr family  Transcriptional regulator, XRE family Transcriptional regulator, ArsR family Transcriptional regulator, WhiB family Transcriptional regulator  RNA polymerase sigma factor RpoD RNA polymerase principal sigma factor HrdD  RNA polymerase sigma-54 factor RpoN | | | | **57 coding sequences**  Transcriptional regulator, MerR family Periplasmic binding protein/LacI transcriptional regulator  Transcriptional regulator, PadR family Transcriptional regulator, ArsR family Transcriptional regulator, AcrR family Transcriptional regulator, AcrR family Transcriptional regulator, LacI family Transcriptional regulator, AcrR family Transcriptional regulator, PadR family Transcriptional regulator, ArsR family Transcriptional regulator, MarR family Transcriptional regulator, GntR family Transcriptional regulator, AcrR family Transcriptional regulator, ArsR family Transcriptional regulator, LacI family Transcriptional regulator, MerR family Transcriptional regulator, AcrR family Transcriptional regulator, MarR family Transcriptional regulator, IclR family Transcriptional regulator, Xre family Ribonucleotide reductase transcriptional regulator NrdR  Transcriptional regulator  Transcriptional regulator, LacI family Transcriptional regulator, AcrR family Probable transcriptional regulatory protein YebC T  Transcriptional regulator, AcrR family Putative AsnC-family transcriptional regulatory protein  Nudix-related transcriptional regulator NrtR  FIG005453: Putative DeoR-family transcriptional regulator  Transcriptional regulator, GntR family Transcriptional regulator of pyridoxine metabolism / Pyridoxamine phosphate aminotransferase (EC 2.6.1.54) Transcriptional regulator, AsnC family Transcriptional regulator MarR Transcriptional regulator, LysR family Transcriptional regulator, AcrR family Transcriptional regulator, AcrR family transcriptional regulatory protein PadR- Transcriptional regulator, AcrR family Transcriptional regulator, ArsR family CarD-like transcriptional regulator Phosphate regulon transcriptional regulatory protein PhoB (SphR)  Putative TetR-family transcriptional regulator  Transcriptional regulator, AcrR family Transcriptional regulator, WhiB family Transcriptional regulator Mn-dependent transcriptional regulator MntR Transcriptional regulator, MarR family Respiratory nitrate reductase gamma chain (EC 1.7.99.4) / Transcriptional regulator, ArsR family / Molybdopterin synthase sulfur carrier subunit Respiratory nitrate reductase gamma chain (EC 1.7.99.4) / Transcriptional regulator, ArsR family / Molybdopterin synthase sulfur carrier subunit Transcriptional regulator, AcrR family Transcriptional regulator, ArsR family Transcriptional regulator, MerR family Transcriptional regulator, GntR family Transcriptional regulator, DeoR family  RNA polymerase principal sigma factor HrdD  RNA polymerase sigma factor RpoD RNA polymerase ECF-type sigma factor | | | | **61 coding sequences**  Transcriptional regulator, MerR family Transcriptional regulator, DeoR family Transcriptional regulator, ArsR family Transcriptional regulator, AcrR family Transcriptional regulator, MarR family Transcriptional regulator, AcrR family Transcriptional regulator  Transcriptional regulator, AcrR family Transcriptional regulator, PadR family Transcriptional regulator, ArsR family Transcriptional regulator, MarR family Transcriptional regulator, GntR family Transcriptional regulator, AcrR family Transcriptional regulator, ArsR family Transcriptional regulator, LacI/PurR family  Transcriptional regulator, MerR family Transcriptional regulator, AcrR family Transcriptional regulator, MarR family Transcriptional regulator, AcrR family Nudix-related transcriptional regulator NrtR  Transcriptional regulator, AsnC family Transcriptional regulator, AcrR family Probable transcriptional regulatory protein YebC  Transcriptional regulator, AcrR family Transcriptional regulator, LacI family Transcriptional regulator  Ribonucleotide reductase transcriptional regulator NrdR  Transcriptional regulator, Xre family Transcriptional regulator, AcrR family Transcriptional regulator, IclR family FIG005453: Putative DeoR-family transcriptional regulator  Transcriptional regulator, GntR family Transcriptional regulator of pyridoxine metabolism / Pyridoxamine phosphate aminotransferase (EC 2.6.1.54) Transcriptional regulator MraZ Transcriptional regulator, AsnC family Transcriptional regulator MarR Transcriptional regulator, LysR family Transcriptional regulator, AcrR family Transcriptional regulator, AcrR family transcriptional regulatory protein PadR- Transcriptional regulator, AcrR family Transcriptional regulator, ArsR family Transcriptional regulator, MarR family CarD-like transcriptional regulator Phosphate regulon transcriptional regulatory protein PhoB (SphR) Transcriptional regulator, AcrR family Transcriptional regulator, AcrR family Transcriptional regulator, WhiB family Mn-dependent transcriptional regulator MntR  Transcriptional regulator, MarR family Respiratory nitrate reductase gamma chain (EC 1.7.99.4) / Transcriptional regulator, ArsR family / Molybdopterin synthase sulfur carrier subunit Transcriptional regulator, AcrR family Transcriptional regulator, ArsR family Transcriptional regulator, MerR family Transcriptional regulator, GntR family Transcriptional regulator, PadR family Transcriptional regulator, LacI family  RNA polymerase ECF-type sigma factor RNA polymerase ECF-type sigma factor RNA polymerase sigma factor RpoD RNA polymerase principal sigma factor | | | |
| Heat shock | **11 coding sequences**  Heat shock protein 60 family co-chaperone GroES  Heat shock protein 60 family chaperone GroEL  Hypothetical radical SAM family enzyme in heat shock gene cluster, similarity with CPO of BS HemN-type Heat-inducible transcription repressor HrcA  Heat shock protein 60 family chaperone GroEL  Heat shock protein GrpE  COG0071: Molecular chaperone (small heat shock protein)  Heat shock protein 22.5 (Hsp22.5)  Heat shock protein 60 family co-chaperone GroES  Ribosome-associated heat shock protein implicated in the recycling of the 50S subunit (S4 paralog)  COG0071: Molecular chaperone (small heat shock protein) | | | | **13 coding sequences**  Heat shock protein GrpE  Heat shock protein 60 family co-chaperone GroES  Heat shock protein 60 family chaperone GroEL  Heat shock protein 60 family co-chaperone GroES  Hypothetical radical SAM family enzyme in heat shock gene cluster, similarity with CPO of BS HemN-type Heat-inducible transcription repressor HrcA COG0071: Molecular chaperone (small heat shock protein)  Heat shock protein GrpE  Heat shock protein 22.5 (Hsp22.5)  Heat shock protein 60 family chaperone GroEL  Ribosome-associated heat shock protein implicated in the recycling of the 50S subunit (S4 paralog)  COG0071: Molecular chaperone (small heat shock protein)  Heat shock protein Hsp20 | | | | **11 coding sequences**  Heat shock protein GrpE  Heat shock protein 10 kDa family chaperone GroES  Heat shock protein 60 kDa family chaperone GroEL  Heat-inducible transcription repressor HrcA  COG0071: Molecular chaperone (small heat shock protein)  Heat shock protein GrpE  Heat shock protein 22.5 (Hsp22.5)  Heat shock protein 60 kDa family chaperone GroEL  Ribosome-associated heat shock protein implicated in the recycling of the 50S subunit (S4 paralog)  COG0071: Molecular chaperone (small heat shock protein)  Heat shock protein Hsp20 | | | |
| Cold shock | **2 coding sequences**  Cold shock protein of CSP family  Cold shock protein of CSP family => SCO4325 | | | | **2 coding sequences**  Cold shock protein of CSP family  Cold shock protein of CSP family => SCO4325 | | | | **2 coding sequences**  Cold shock protein of CSP family  Cold shock protein of CSP family => SCO4325 | | | |
| Chaperone | **12 coding sequences**  Heat shock protein 60 family co-chaperone GroES  Heat shock protein 60 family chaperone GroEL PFIG00823557: AC2 (Proteasome assembly chaperone) family  Chaperone protein DnaJ  Heat shock protein 60 family chaperone GroEL Copper(I) chaperone CopZ  Chaperone protein DnaJ  Chaperone protein DnaK  COG0071: Molecular chaperone (small heat shock protein)  Heat shock protein 60 family co-chaperone GroES  COG2608: Copper chaperone  COG0071: Molecular chaperone (small heat shock protein) | | | | **16 coding sequences**  Chaperone protein DnaK  Chaperone protein DnaJ  Heat shock protein 60 family co-chaperone GroES  Heat shock protein 60 family chaperone GroEL Heat shock protein 60 family co-chaperone GroES  Chaperone protein DnaJ  COG0071: Molecular chaperone (small heat shock protein)  Chaperone protein DnaJ  Chaperone protein DnaK  Heat shock protein 60 family chaperone GroEL Anaerobic dimethyl sulfoxide reductase chaperone DmsD  Zn-dependent protease with chaperone function Copper(I) chaperone CopZ  COG0071: Molecular chaperone (small heat shock protein)  COG2608: Copper chaperone  Inner membrane protein translocase and chaperone YidC, short form OxaI-like | | | | **16 coding sequences**  Chaperone protein DnaK  Chaperone protein DnaJ  Heat shock protein 10 kDa family chaperone GroES Heat shock protein 60 kDa family chaperone GroEL  Chaperone protein DnaJ  COG0071: Molecular chaperone (small heat shock protein)  Chaperone protein DnaJ  Chaperone protein DnaK  Chaperone protein ClpB (ATP-dependent unfoldase)  Heat shock protein 60 kDa family chaperone GroEL  Chaperone protein ClpB (ATP-dependent unfoldase)  Asenic metallochaperone ArsD, transfers trivalent metalloids to ArsAB pump  Anaerobic dimethyl sulfoxide reductase chaperone DmsD  Copper(I) chaperone CopZ  COG0071: Molecular chaperone (small heat shock protein)  Inner membrane protein translocase and chaperone YidC, short form OxaI-like | | | |
| Oxidative stress | **16 coding sequences**  Thioredoxin  Thioredoxin  Thioredoxin reductase (EC 1.8.1.9)  FIG000875: thioredoxin domain-containing protein EC-YbbN  Thioredoxin  Thioredoxin  Phosphoadenylyl-sulfate reductase [thioredoxin] (EC 1.8.4.8)  Thioredoxin reductase (EC 1.8.1.9)  Catalase KatE (EC 1.11.1.6)  Thiol peroxidase, Bcp-type (EC 1.11.1.15) Dyp-type peroxidase  Thiol peroxidase, Tpx-type (EC 1.11.1.15)  Superoxide dismutase [Mn/Fe] (EC 1.15.1.1)  Glutathione S-transferase, omega (EC 2.5.1.18)  Glutathione S-transferase, omega (EC 2.5.1.18) Cysteine synthase (EC 2.5.1.47)  Cysteine synthase (EC 2.5.1.47) | | | | **18 coding sequences**  Thioredoxin  Thioredoxin reductase (EC 1.8.1.9) FIG000875: Thioredoxin domain-containing protein EC-YbbN  Thioredoxin 2  Thioredoxin  Thioredoxin  Arsenate reductase (EC 1.20.4.4) thioredoxin-coupled, LMWP family Phosphoadenylyl-sulfate reductase [thioredoxin] (EC 1.8.4.8)  Thioredoxin reductase (EC 1.8.1.9) Thioredoxin  Catalase KatE (EC 1.11.1.6)  Thiol peroxidase, Tpx-type (EC 1.11.1.15) Thiol peroxidase, Bcp-type (EC 1.11.1.15) Dyp-type peroxidase  Superoxide dismutase [Mn/Fe] (EC 1.15.1.1)  Putative glutathione S-transferase (EC 2.5.1.18)  Glutathione S-transferase, omega (EC 2.5.1.18) Cysteine synthase (EC 2.5.1.47)  Cysteine synthase (EC 2.5.1.47) | | | | **17 coding sequences**  Thioredoxin  Thioredoxin reductase (EC 1.8.1.9) FIG000875: Thioredoxin domain-containing protein EC-YbbN Thioredoxin 2  Thioredoxin  Thioredoxin  Arsenate reductase (EC 1.20.4.4) thioredoxin-coupled, LMWP family Phosphoadenylyl-sulfate reductase [thioredoxin] (EC 1.8.4.8)  Thioredoxin reductase (EC 1.8.1.9) Thioredoxin  Catalase KatE (EC 1.11.1.6)  Thiol peroxidase, Tpx-type (EC 1.11.1.15) Thiol peroxidase, Bcp-type (EC 1.11.1.15) Dyp-type peroxidase  Superoxide dismutase [Mn/Fe] (EC 1.15.1.1)  Glutathione S-transferase, omega (EC 2.5.1.18) Cysteine synthase (EC 2.5.1.47)  Cysteine synthase (EC 2.5.1.47) | | | |
| Toxic environment | **3 coding sequences**  Multidrug ABC transporter, permease protein  Arsenical pump-driving ATPase (EC 3.6.3.16)  Heavy-Metal transporting ATPase | | | | **3 coding sequences**  Small multidrug resistance family (SMR) protein  Arsenical pump-driving ATPase (EC 3.6.3.16) TEMP  Heavy-Metal transporting ATPase | | | | **3 coding sequences**  Small multidrug resistance family (SMR) protein  Arsenite/antimonite pump-driving ATPase ArsA (EC 3.6.3.16)  Heavy-Metal transporting ATPase | | | |
| Intracellular pH | **19 coding sequences**  ATP synthase F0 sector subunit a (EC 3.6.3.14) ATP synthase F0 sector subunit c (EC 3.6.3.14) ATP synthase F0 sector subunit b (EC 3.6.3.14) ATP synthase delta chain (EC 3.6.3.14)  ATP synthase alpha chain (EC 3.6.3.14)  ATP synthase gamma chain (EC 3.6.3.14)  ATP synthase beta chain (EC 3.6.3.14)  ATP synthase epsilon chain (EC 3.6.3.14)  Pyruvate-flavodoxin oxidoreductase (EC 1.2.7.-)  Pyruvate-flavodoxin oxidoreductase (EC 1.2.7.-)  Aspartate ammonia-lyase (EC 4.3.1.1)  Aspartate ammonia-lyase (EC 4.3.1.1)  Succinate dehydrogenase cytochrome b subunit Succinate dehydrogenase flavoprotein subunit (EC 1.3.5.1)  Succinate dehydrogenase iron-sulfur protein (EC 1.3.5.1)  Succinate dehydrogenase cytochrome b subunit Succinate dehydrogenase flavoprotein subunit (EC 1.3.5.1)  Succinate dehydrogenase iron-sulfur protein (EC 1.3.5.1)  Glutamate decarboxylase (EC 4.1.1.15) | | | | **19 coding sequences**  ATP synthase F0 sector subunit a (EC 3.6.3.14)  ATP synthase F0 sector subunit c (EC 3.6.3.14) ATP synthase F0 sector subunit b (EC 3.6.3.14) ATP synthase delta chain (EC 3.6.3.14)  ATP synthase alpha chain (EC 3.6.3.14)  ATP synthase gamma chain (EC 3.6.3.14)  ATP synthase beta chain (EC 3.6.3.14)  ATP synthase epsilon chain (EC 3.6.3.14)  Pyruvate-flavodoxin oxidoreductase (EC 1.2.7.-)  Pyruvate-flavodoxin oxidoreductase (EC 1.2.7.-)  Aspartate ammonia-lyase (EC 4.3.1.1)  Aspartate ammonia-lyase (EC 4.3.1.1)  Succinate dehydrogenase iron-sulfur protein (EC 1.3.5.1)  Succinate dehydrogenase flavoprotein subunit (EC 1.3.5.1)  Succinate dehydrogenase cytochrome b subunit Succinate dehydrogenase iron-sulfur protein (EC 1.3.5.1)  Succinate dehydrogenase flavoprotein subunit (EC 1.3.5.1)  Succinate dehydrogenase cytochrome b subunit  Glutamate decarboxylase (EC 4.1.1.15) | | | | **19 coding sequences**  ATP synthase F0 sector subunit a (EC 3.6.3.14)  ATP synthase F0 sector subunit c (EC 3.6.3.14)  ATP synthase F0 sector subunit b (EC 3.6.3.14)  ATP synthase delta chain (EC 3.6.3.14)  ATP synthase alpha chain (EC 3.6.3.14)  ATP synthase gamma chain (EC 3.6.3.14)  ATP synthase beta chain (EC 3.6.3.14)  ATP synthase epsilon chain (EC 3.6.3.14)  Pyruvate-flavodoxin oxidoreductase  Pyruvate-flavodoxin oxidoreductase  Aspartate ammonia-lyase (EC 4.3.1.1)  Aspartate ammonia-lyase (EC 4.3.1.1)  Succinate dehydrogenase cytochrome b subunit Succinate dehydrogenase flavoprotein subunit (EC 1.3.5.1)  Succinate dehydrogenase iron-sulfur protein (EC 1.3.5.1)  Succinate dehydrogenase iron-sulfur protein (EC 1.3.5.1)  Succinate dehydrogenase flavoprotein subunit (EC 1.3.5.1)  Succinate dehydrogenase cytochrome b subunit  Glutamate decarboxylase (EC 4.1.1.15) | | | |
| Osmotic stress | **7 coding sequences**  Glycine betaine/L-proline transport ATP-binding protein ProV (TC 3.A.1.12.1)  L-Proline/Glycine betaine transporter ProP  L-Proline/Glycine betaine transporter ProP Glycine betaine/L-proline transport ATP-binding protein ProV (TC 3.A.1.12.1)  Glycine betaine ABC transport system permease protein  L-Proline/Glycine betaine transporter ProP Glycine betaine/L-proline transport substrate-binding protein ProX (TC 3.A.1.12.1) | | | | **2 coding sequnces**  L-Proline/Glycine betaine transporter ProP  L-Proline/Glycine betaine transporter ProP | | | | **2 coding sequnces**  L-Proline/Glycine betaine transporter ProP  L-Proline/Glycine betaine transporter ProP | | | |
| Alarmone ppGpp | **5 coding sequences**  Polyribonucleotide nucleotidyltransferase (EC 2.7.7.8)  Inosine-5'-monophosphate dehydrogenase (EC 1.1.1.205) / CBS domain  Inosine-5'-monophosphate dehydrogenase (EC 1.1.1.205) / CBS domain  Inosine-5'-monophosphate dehydrogenase, catalytic domain (EC 1.1.1.205)  Inosine-uridine preferring nucleoside hydrolase (EC 3.2.2.1) | | | | **5 coding sequences**  Polyribonucleotide nucleotidyltransferase (EC 2.7.7.8)  Inosine-5'-monophosphate dehydrogenase (EC 1.1.1.205) / CBS domain  Inosine-5'-monophosphate dehydrogenase, catalytic domain (EC 1.1.1.205)  Inosine-5'-monophosphate dehydrogenase (EC 1.1.1.205) / CBS domain  Inosine-uridine preferring nucleoside hydrolase (EC 3.2.2.1) | | | | **5 coding sequences**  Polyribonucleotide nucleotidyltransferase (EC 2.7.7.8)  Inosine-5'-monophosphate dehydrogenase (EC 1.1.1.205) / CBS domain  Inosine-5'-monophosphate dehydrogenase, catalytic domain (EC 1.1.1.205)  Inosine-5'-monophosphate dehydrogenase (EC 1.1.1.205) / CBS domain  Inosine-uridine preferring nucleoside hydrolase (EC 3.2.2.1) | | | |
| polyP | **+** | | | | **+** | | | | **+** | | | |
| Trehalose | **+** | | | | **+** | | | | **+** | | | |
| Glycogen | **+** | | | | **+** | | | | **+** | | | |

based on RAST
