# Supplementary figures and images for: Sequencing and Analysis of the Genome of Propionibacterium freudenreichii T82 Strain: Importance for Industry
Source: Biomolecules. 2020 Feb 24;10(2):348. doi: 10.3390/biom10020348 (PMC7072396; doi:10.3390/biom10020348)

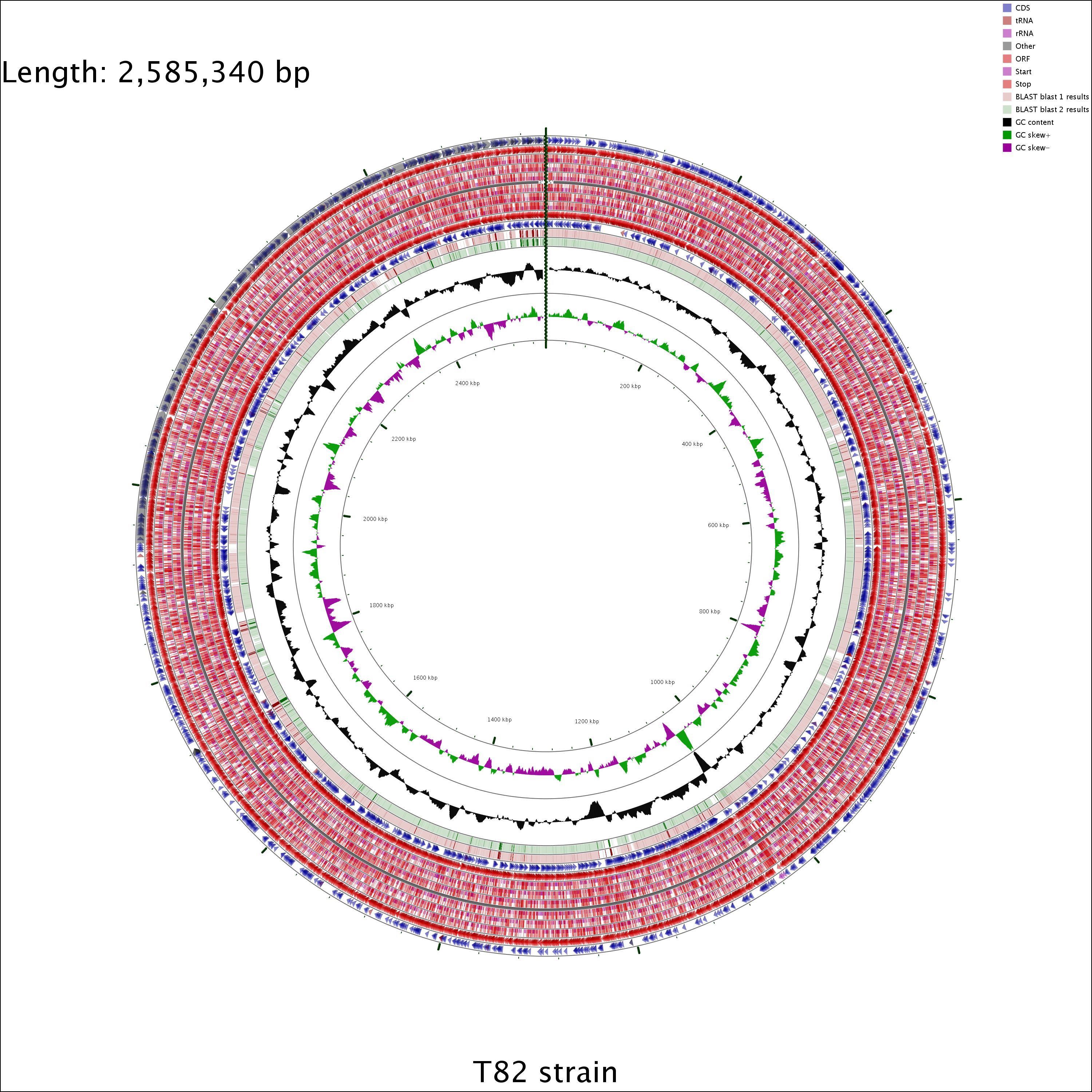

Supplement: Supplementary file 1 [file biomolecules-10-00348-s001.zip › Supplementary materials/Figure S11 P. freudenreichii T82.png]
